# A case of incomplete Kawasaki disease with extremely high serum ferritin and interleukin-18 levels

**DOI:** 10.1186/s12887-018-1365-7

**Published:** 2018-12-12

**Authors:** Takanori Noto, Hiroki Seto, Junji Fukuhara, Masao Murabayashi, Akihiro Yachie, Mamoru Ayusawa, Ichiro Morioka

**Affiliations:** 1 Department of Pediatrics, Numazu City Hospital, 550, Harunoki, Higashi-Shiroji, Numazu, 4100302 Japan; 2 Department of Pediatrics and Child Health, Nihon University School of Medicine, 30-1, Oyaguchi, Kami-cho, Itabashi-ku, Tokyo, 173-8610 Japan; 3 Department of Pediatrics, School of Medicine, Institute of Medical, Pharmaceutical and Health Sciences, Kanazawa University, 13-1, Takaramachi, Kanazawa, 9208641 Japan

**Keywords:** Ferritin, Interleukin-6, Interleukin-18, Kawasaki disease, Systemic juvenile idiopathic arthritis

## Abstract

**Background:**

The clinical features and laboratory parameters of patients with Kawasaki disease (KD) and systemic juvenile idiopathic arthritis (sJIA) occasionally overlap. Therefore, serum levels of cytokine and ferritin are used as markers to distinguish between KD and sJIA. KD patients have a high level of interleukin (IL)-6, low level of IL-18, and no elevation of the level of serum ferritin. Conversely, sJIA patients have a low level of IL-6 and high levels of IL-18 and ferritin in the serum. However, to the best of our knowledge, no case report of KD with a low serum level of IL-6 and extremely high levels of IL-18 and ferritin is found.

**Case presentation:**

A 6-year-old boy presented with a history of fever for 9 days and a rash that appeared 7 days from the onset. He was diagnosed with incomplete KD because of fever, skin rash, oral cavity erythematous changes, and erythema and edema of the hands with laboratory findings of serum albumin level < 3.0 g/dL, elevated alanine aminotransferase level and leukocyturia. Intravenous immunoglobulin and prednisolone and oral aspirin were introduced on the 10th day. Fever subsided 1 day after initiating the treatment, but arthritis of both knees appeared in addition to hepatosplenomegaly. We suspected sJIA, as the serum level of ferritin was 19,740 ng/mL, IL-6 was < 3 pg/mL, and IL-18 was 132,000 pg/mL. Skin desquamation of the fingertips was observed 18 days from the onset; thus, he was finally diagnosed with incomplete KD with arthritis. At 32 days from the onset, we stopped the prednisolone therapy and no symptoms of relapse were observed afterwards. In the follow-up at 16 months from the onset, he had neither signs of active joint or skin involvement, nor cardiac involvement.

**Conclusions:**

Although patients with sJIA generally have high serum levels of IL-18 and ferritin, this was a case of incomplete KD with extremely high serum levels of IL-18 and ferritin. Serum cytokine and ferritin are often used for the differential diagnosis of KD and sJIA. We need to recognize the existence of KD with high serum levels of IL-18 and ferritin.

## Background

Classic Kawasaki disease (KD) is clinically diagnosed based on the five following symptoms: the continuity of fever for at least 5 days, oral cavity erythematous changes (cracked lips, strawberry tongue), bilateral bulbar conjunctival injection, skin rash (maculopapular, diffuse erythroderma, or erythema multiforme-like), erythema and edema of the hands and feet, and cervical lymphadenopathy (usually unilateral) [1]. Incomplete KD has also been known as one type of KD, which is diagnosed based on fever more than 5 days with two or three of the five aforementioned symptoms or fever for ≥7 days without other explanations, and compatible laboratory or echocardiographic findings [1, 2]. Desquamation of the periungual region in the fingers usually begins in 2 to 3 weeks after the onset of fever. Some patients with KD develop arthritis, and the frequency of this condition is reported as 7.5–16% [2–4]. Systemic juvenile idiopathic arthritis (sJIA) is characterized by remitting fever, a typical skin rash, and arthritis. Diagnosis of sJIA is often challenging, particularly before patients have the symptoms for 6 weeks as shown by the International League of Associations for Rheumatology and American College of Rheumatology criteria [5]. Given that the clinical features, especially in arthritis, in patients with incomplete KD and sJIA sometimes overlap, cytokines [6–10] and ferritin [11] in serum are used as markers to distinguish KD from sJIA. KD patients have a high interleukin (IL)-6 level and low IL-18 and ferritin levels in their serum. Conversely, sJIA patients have a low IL-6 level and high IL-18 and ferritin levels [6–11]. Contrary to these facts, however, we report for the first time a case of incomplete KD with a low serum IL-6 level and extremely high serum IL-18 and ferritin levels with written informed consent from the parents of the patient.

## Case presentation

A 6-year-old boy was referred to our hospital due to a 9-day history of fever. On day 3 of illness, a diffuse maculopapular rash appeared. He was orally treated with cefcapene pivoxil prescribed by his family pediatrician. On admission, his weight and height were 24 kg and 124 cm, respectively. He had a temperature of 38.9 °C and had a diffuse maculopapular rash. His lips, hands, and feet were erythematous. In addition, he also developed hepatosplenomegaly and had pitting edema in his feet. There was no history or findings of conjunctival injection and cervical lymphadenopathy.

**Fig. 1:**
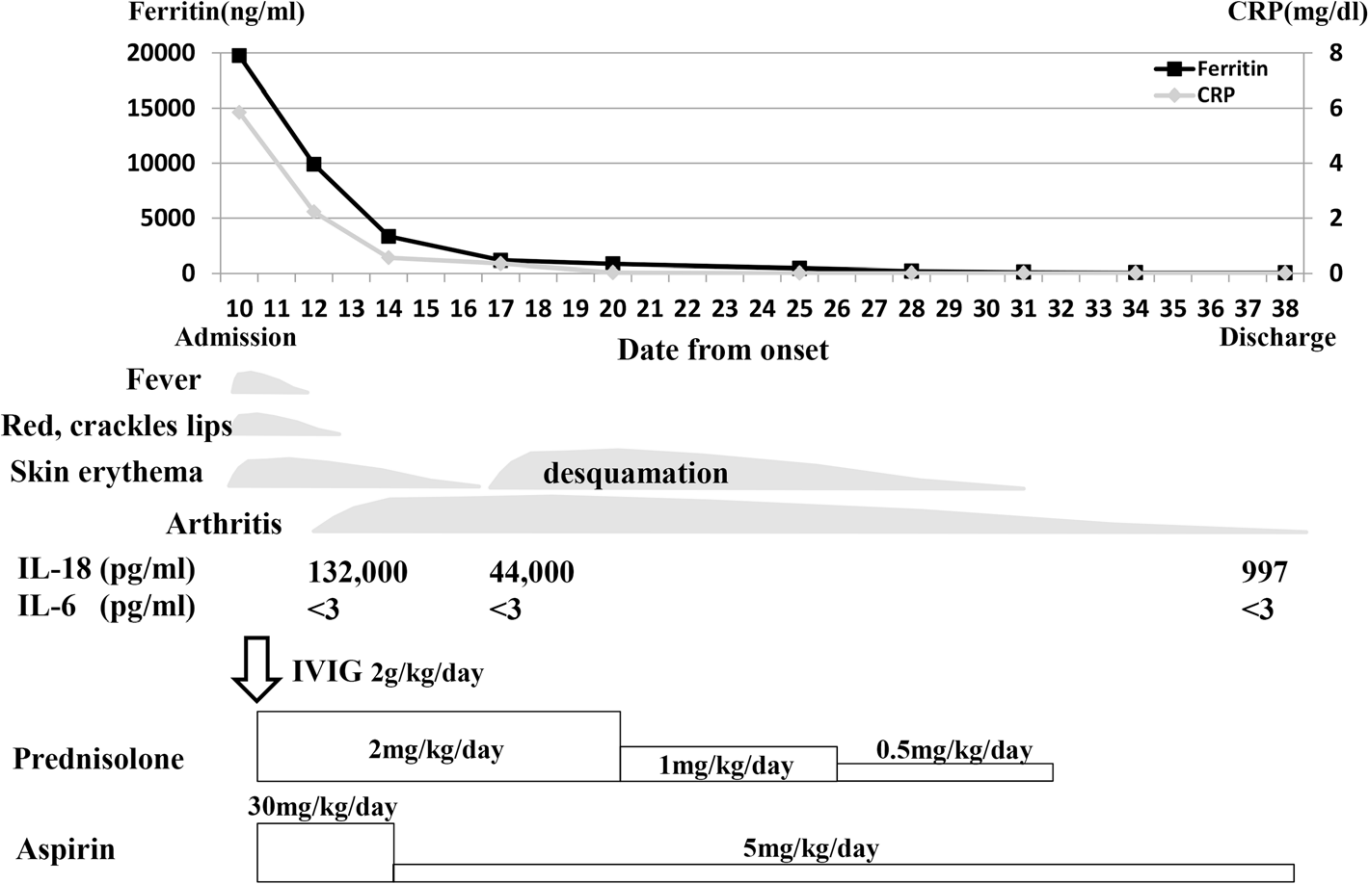
Clinical course. CRP, C-reactive protein; IL, interleukin; IVIG, intravenous immunoglobulin

His clinical course is shown in Fig. 1. Blood examination revealed the following: white blood cell count of 12,800/μL (neutrophils, lymphocytes, and monocytes were 88, 9, and 3%, respectively). C-reactive protein of 5.85 mg/dL; hemoglobin level of 11.7 g/dL; and platelet count of 26.6 × 10^4^/μL. Other blood findings were as follows: serum albumin of 2.7 g/dL, total bilirubin of 0.7 mg/dL, sodium of 131 mEq/L, potassium of 3.1 mEq/L, aspartate aminotransferase of 100 IU/L, alanine aminotransferase of 87 IU/L, lactate dehydrogenase of 613 IU/L, and ferritin of 19,740 ng/mL. His urinalysis showed leukocyturia without any bacteria (10–14 white blood cells/high power field). His chest radiograph showed normal findings. Echocardiography revealed a normal ejection fraction, but perivascular echo brightness of the coronary arteries was found.

Given that his clinical symptoms did not fulfill the diagnostic criteria for classic KD, he was diagnosed with an incomplete KD according to the American Heart Association guideline [2]. Therefore, intravenous immunoglobulin (IVIG, 2 g/kg/dose), intravenous prednisolone (PSL, 2 mg/kg/day), and oral aspirin (30 mg/kg/day) were administered on the 10th day of illness. In addition to IVIG, PSL was used for his treatments to prevent coronary artery abnormalities based on the result of RAISE study [12]. His temperature returned to normal soon after the first IVIG therapy was completed. On the 12th day of illness, however, he showed symptoms of arthritis in both knee joints, which led to a gait disturbance. sJIA was suspected based on the appearance of arthritis and an extremely high level of serum ferritin. To distinguish KD from sJIA, serum IL-6 and IL-18 levels were examined during that time. The serum levels of IL-6 and IL-18 were extremely low and high, respectively (Fig. 1 and Table 1). Given the markedly elevated serum ferritin and IL-18 levels, we suspected sJIA rather than incomplete KD. However, his rheumatoid factor and anti-nuclear antibody were negative. On the 17th day of illness, the elevated serum IL-18 level persisted (44,000 pg/mL) and his arthritis worsened. On the 18th day of illness, because skin desquamation of the fingertips occurred, he was diagnosed with incomplete KD. After the PSL treatment of 2 mg/kg/day for 11 days, PSL was tapered to 1 mg/kg/day for 5 days and then 0.5 mg/kg/day for 5 days. On the 32nd day of illness, the PSL treatment was discontinued, as his joint symptoms were markedly improved. After the aspirin treatment of 30 mg/kg/day for 5 days, aspirin of 5 mg/kg/day was discontinued after 2 months of the disease onset. On the 116th day of illness, serum IL-18 level returned to normal. At 16 months after the disease onset, he had never shown any signs of joint or skin involvement and cardiac abnormalities including coronary arteries.

**Table 1:** Serum ferritin, IL-18, and IL-6 levels of our case and the reference levels

	Our case	KD	sJIA
Ferritin, ng/mL	19,740	14–2376	63–68,310
IL-18, pg/mL	132,000	260–660	10,860–330,000
IL-6, pg/mL	< 3	106–1200	7–580

The reference values of ferritin in KD and sJIA are from Mizuta et al. [11] and those of IL-18 and IL-6 are from Takahara et al. [7]

*IL* interleukin, *KD* Kawasaki disease, *sJIA* systemic juvenile idiopathic arthritis

## Discussion and conclusions

The clinical features and laboratory findings in some patients with incomplete KD and other systemic inflammatory diseases such as macrophage activation syndrome or sJIA overlap. No cytopenia, hypertriglyceridemia and hypofibrinogenemia were found in his clinical course (serum triglyceride level of 217 mg/dL and plasma fibrinogen level of 323 mg/dL). There was no elevation of serum soluble interleukin-2 receptor level (344 U/mL). Based on these findings, because hemophagocytic lymphohistiocytosis was not suspected, a bone marrow examination was not performed for him. However, it has been reported that the patients who were considered as having refractory KD were finally diagnosed with sJIA [13]. Therefore, some serum markers have been proposed to distinguish KD from sJIA. Mizuta et al. reported that the serum ferritin level was significantly higher in sJIA patients than in KD patients, for which the cutoff value was 368.6 ng/mL [11]. In cytokines, the characteristics of serum markers in sJIA patients include significantly higher levels of IL-18 and lower levels of IL-6 than those in KD patients [6, 7, 9, 10]. In our case, because extremely high serum ferritin and IL-18 levels, unelevated IL-6 levels, and arthritic symptoms were observed during the clinical course (Table 1), sJIA was suspected. However, we finally diagnosed the patient with incomplete KD accompanied with arthritis, because of the following reasons. First, perivascular echo brightness of the coronary arteries was found. Second, a periungual desquamation was observed during the recovery phase (Fig. 1). Third, his arthritis improved within 6 weeks, and the sJIA criteria were not completely fulfilled [14]. Fourth, the IVIG treatment was effective. Fifth, the disease improved during PSL treatment, and no relapse was found after the dose of PSL was tapered. Finally, his clinical symptoms were different from those of sJIA, such as flares of clinical signs and intermittent fever with rash. He looked so sick and agitated during the disease period, although patients with sJIA look almost normal during the intermittent afebrile period.

In conclusion, patients with sJIA generally have high serum IL-18 and ferritin levels [6–11]. This was a case of incomplete KD with extremely high serum levels of IL-18 and ferritin, although KD patients with coronary artery lesions have been reported to have mild IL-18 elevations [15]. Serum cytokines and ferritin are often used for the differential diagnosis of KD and sJIA. However, we should need to recognize the existence of KD in patients with high serum IL-18 and ferritin levels. Further studies are needed about the novel biomarkers to clearly distinguish sJIA from KD at an early phase of the disease progression.

## Abbreviations

IL: Interleukin
IVIG: Intravenous immunoglobulin
KD: Kawasaki disease
PSL: Prednisolone
sJIA: systemic juvenile idiopathic arthritis
